# *Manilkara zapota* “chicozapote” as a fruit source of health-beneficial bioactive compounds and its effects on chronic degenerative and infectious diseases, a review

**DOI:** 10.3389/fnut.2023.1194283

**Published:** 2023-07-04

**Authors:** Maria Fernanda Rivas-Gastelum, Luis Eduardo Garcia-Amezquita, Rebeca Garcia-Varela, Angélica Lizeth Sánchez-López

**Affiliations:** Tecnologico de Monterrey, Escuela de Ingeniería y Ciencias, Zapopan, Jalisco, Mexico

**Keywords:** chicozapote, bioactivity, phytotherapeutics, extraction methodologies, health beneficial applications

## Abstract

*Manilkara zapota* “chicozapote” is an autochthonous evergreen tree from the Southern regions of Mexico, Belize, and Guatemala. Currently, it is widely distributed and extensively grown in Mexico and Southeast Asia. Traditionally, different structures of the plant have been used for medical purposes; seeds have diuretic and purgative properties, aiding in digestive complications and eliminating bladder and kidney stones. Tree bark has antidiarrheal, antipyretic, antibiotic, and astringent properties. Fruits and leaves have been used to treat cold, cough, diarrhea, indigestion, fever, hemorrhages, wounds, and ulcers. Chicozapote fruit is yellow and brown, with an oval shape and rough peel, it is an excellent source of nutrients, such as sugars, proteins, amino acids, and minerals, and is rich in phytochemical components, such as flavonoids, phenolic acids, and tannins. These bioactive compounds exert several biological activities, i.e., as an antioxidant, antidiabetic, antimicrobial, anti-inflammatory, cytotoxic, and anti-arthritic agents, to name a few. These beneficial properties assist in preventing chronic and degenerative diseases, such as cancer, diabetes, neurological, infectious, and cardiovascular diseases. The use of chicozapote is still limited to its fresh form, and its non-edible structures produce a lot of waste. Therefore, an alternative valorizing and preserving strategy is to use the fruit as a raw source to design functional foods and pharmacological products. Here, the nutritional and phytochemical profiles and the current view regarding methodologies and conditions, for the extraction and characterization of its bioactive compounds, are described, and focus is placed on their multiple biological effects and specific functional mechanisms.

## 1. Introduction

**Figure 1:**
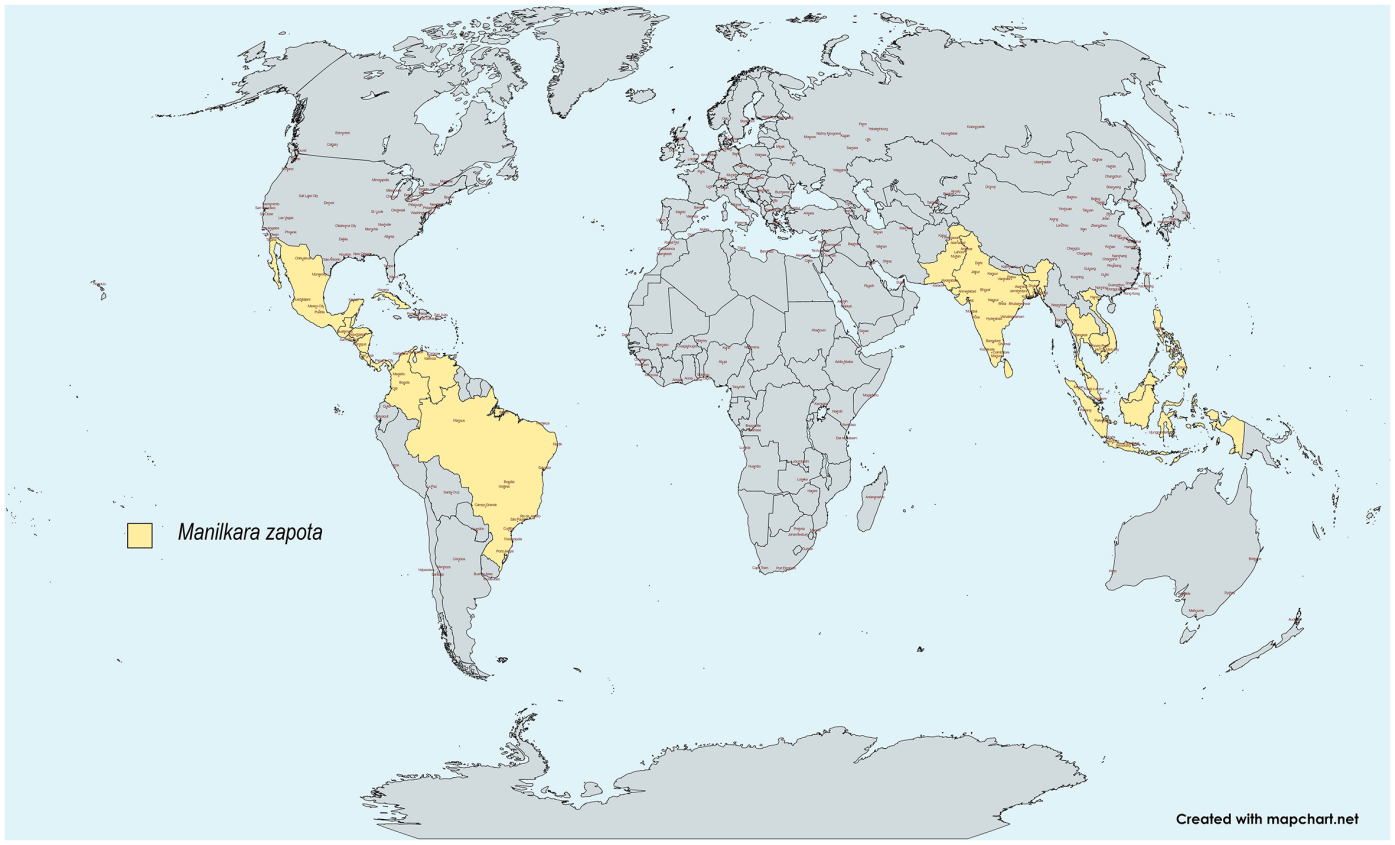
Worldwide distribution of *chicozapote.* Created with Mapchart.net.

*Manilkara zapota (L.)* commonly referred to as “chicozapote,” sapodilla (Mexico), chiku (India), and nispero (Puerto Rico), is an evergreen tree native to the Southern regions of Mexico, Northern Guatemala, and Belize. It is widely distributed, particularly in Central and South America. *Chicozapote* was introduced to Southeast Asia because of its exceptionally sweet and malty flavor. It is extensively cultivated for commercial purposes, especially in The Philippines, Indonesia, Vietnam, Malaysia, India, and Thailand (1–4). It is also abundantly cultivated in Bangladesh, Cambodia, Sri Lanka, and Pakistan (5, 6) (Figure 1). Chicozapote belongs to the *Sapotaceae* family, along with 1,250 flowering species, and is known for its quality wood, medicinal properties, and sticky and often white latex (7–9). Chicozapote is a tree that can reach a height of 25–45 m, with an average diameter of 1.5 m (3). Under tropical conditions, it bears white, bell-like flowers in different flushes (June–July, September–October, and March–April) (5, 6).

**Figure 2:**
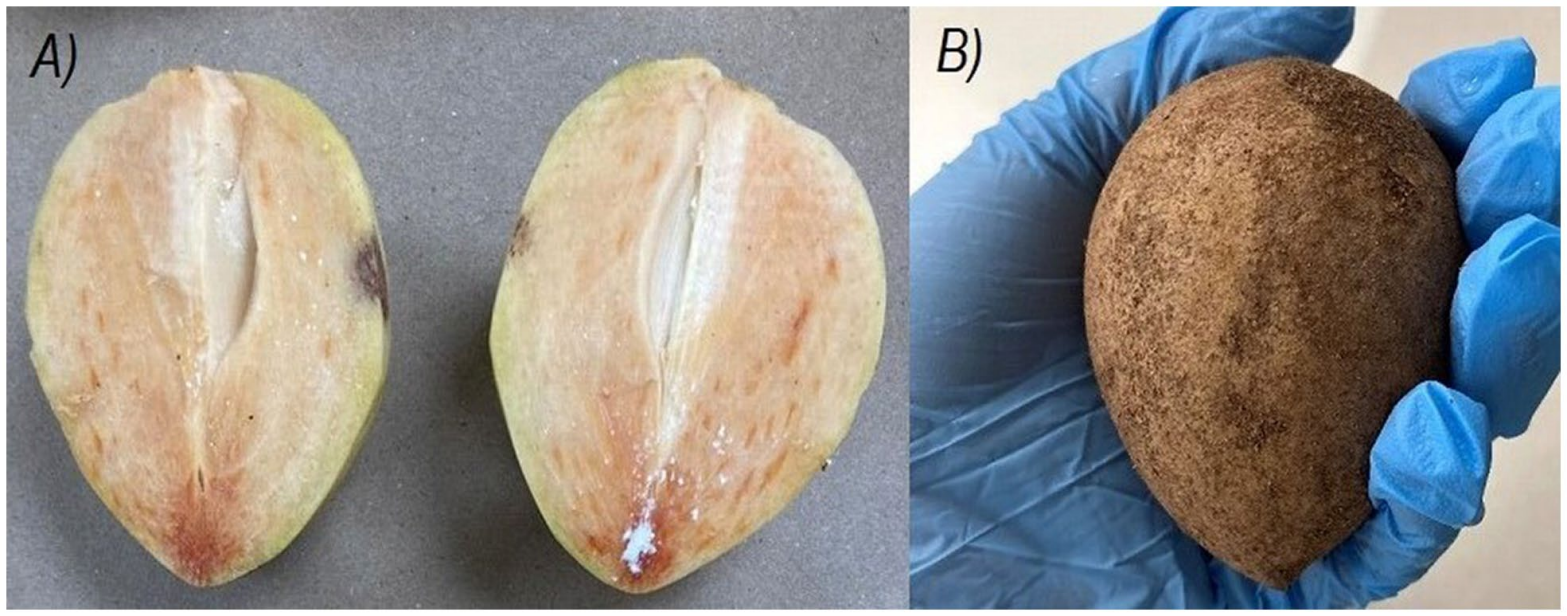
Unripe chicozapote fruit. **(A)** Fruit pericarp by half. **(B)** Fruit from the outside.

The botanical classification of chicozapote fruit is currently a subject of discussion. Several authors assert that it falls within the berry category (10–14) whereas others suggest that it may be more appropriately classified as a drupe (15). The fruit may be round, oblate, or oval; ranges from 5 to 9 cm in diameter and weighs 70–300 g (5). It has a brown rough and gritty peel texture. It has a fleshy and juicy pulp with a characteristic reddish-brown color. When immature, is hard, highly astringent, and gummy due to its latex content (Figure 2) (16, 17). Typically, 1–5 black shiny seeds can be found inside. The pulp, peel, and seed represent 79, 15, and 5% of the total fruit weight, respectively. Moisture content of the entire fruit was estimated at 78%, similar to pulp (80%) (9). The edible portion is about 70%, whereas the non-edible portion accounts for approximately 30% of the total weight (16). The flavor is sweet when mature and resembles the taste profile of pears (8). Traditionally, it has been used to treat cough, diarrhea ulcers, hemorrhage, muscle spasms, pain, pulmonary diseases, and nervous system disorders, among other medical conditions (3, 9). This fruit undergoes climacteric maturation; the commercial maturation stage is reached at 6–9 post-harvest days at room temperature (RT), with a maximum reaching at 11 days (10). Fructification varies according to genotype, climatic conditions, and location (1). However, the main harvest season is between October and February (6). Chicozapote fruit are considered mature enough for harvesting when they become smooth, have a shiny brown color, and have rounded ends (11). Latex is often present in unripe fruits and is absorbed when it reaches physiological maturity. Fruits harvested in premature stages are highly astringent, sweet, and have an alcoholic aftertaste. In contrast, late harvested fruits are soft and have a short shelf life. On average, a single tree can produce 208 kg of fruit annually (12). India, Mexico, Venezuela, and Guatemala are the world’s largest producers (3). Post-harvest management is complex because of its high respiration and ripening rates; thus, its natural pH is close to neutral, making it prone to accelerated microbial spoilage, resulting in a highly perishable product (14). Decay is the main limitation of post-harvest shelf-life, and thus, rapid senescence (5, 13). Although low-temperature storage can extend shelf-life, the fruit is prone to chill injuries at temperatures below 4°C (13); nevertheless, different attempts have been made to extend the post-harvest shelf-life. Applying chemicals, packaging and distribution designs, heat treatments, irradiation, waxing/coating, cold storage, lyophilization, and controlled-atmosphere storage have been explored (18–23). Additionally, the application of different food technologies have led to the development of a range of processed fruit products to be preserved as juice, syrup, ice cream, and wine, to name a few (4, 24–29). Hence, an alternative valorizing and preserving strategy is to use the fruit as a raw source to design functional foods and products with potential health benefits. Here, the phytochemical profile, the current view regarding methodologies and conditions for the extraction, and characterization of chicozapote bioactive compounds are described, focusing on their multiple biological potentials and specific functional mechanisms.

## 2. Nutritional, phytochemical, and aromatic profile

Chicozapote fruit is considered a high-value source of nutrients, minerals, and bioactive phytochemicals (16). According to the United States Department of Agriculture (USDA), the nutritional value of 100 g of chicozapote fruit has been determined; calories (83 Cal), protein (0.4 g), fat (1.1 g), carbohydrates (20 g), and total dietary fiber (5.3 g). Regarding its mineral composition, is high in potassium (193 mg), followed by calcium (21 mg), magnesium (12 mg), and phosphorus (12 mg). A metabolic profile performed by Das and De (26), identified malic, lactic, and succinic acid as the major organic acids of fruit, which are found at all ripening stages, and strongly influence in flavor. Furthermore, is a good source of vitamins, including vitamin A, B complex, C, folate, niacin, and pantothenic acid (6, 16). Sudha and Malarkodi (27) performed a proximate analysis on seven different *Chicozapote* fruit varieties, which resulted in a total soluble solid (TSS) from 17 to 23.40 °Brix, this is considered higher than usual when compared to other fruits. Therefore, the high calorie content is due the high carbohydrate content, which is considered one of the main constituents, along with tannins (3, 6, 8, 21, 23). Chicozapote phytochemical profile is composed mainly of polyphenols, flavonoids, and tannins (Table 1) (8, 28). Tannins include ellagi-tannins and gallotannins (6). Terpenes, alkaloids, saponins, steroids and glycosides are also present (29, 30). Shui et al. (36), reported that chicozapote fruit contains approximately 24 antioxidant compounds. Protocatechuic acid is the most abundant phenolic compound, followed by gallic acid (GA) and quercetin (QUE) (3, 37). Other polyphenolic antioxidants have been isolated from chicozapote, including methyl chlorogenate, myricitrin, (+)-catechin, (−)-epicatechin, (+)-gallocatechin, kaempferol, and dihydromyricetin (7, 38). Novel compounds methyl-4-*O*-galloylcholorogenate and 4-*O*-galloylchlorogenic acid have been identified in chicozapote, both of which have high antioxidant capacity (14). These were first discovered in the methanolic extract of the fruit and were isolated as new antioxidants (32). Whereas Apigenin-7-*O*-β-glucuronide methyl ester (AG) was recently isolated from leaves (39). As its rich in phytochemicals, different values have been reported in literature. Salleh et al. (23), reported a total phenolic content (TPC) of 99.00 ± 12.30 mg of gallic acid equivalent (GAE)/(100g) in fresh pulp. Pravin and Shashikant (38), reported TPC and total flavonoid content (TFC) in peel to be 1151.40 ± 32.3 GAE/(100 g) and 564.50 ± 30.50 quercetin equivalent (QE)/(100 g), respectively. Aromatic compounds in fresh and dried chicozapotes were characterized by gas-chromatography; resulting aromatic schemes were significantly distinct, when fresh they were found to be minty, fatty/green, woody, and spicy. Whereas dried presented a citrusy, balsamic/sweet, and fatty/green profile. The most abundant aromatic components characterized in fresh fruit were ethyl benzoate, methyl benzoate, and hexyl benzoate. In contrast, dried chicozapote was mainly hexyl benzoate, 3-Methy-1-butanol, and ethyl benzoate. Owing to its stabilization during the drying process, 19 aromatic-active compounds were detectable in dried fruit compared to 13 found in fresh fruit (40).

**Table 1:** Chicozapote identified compounds by sections.

Plant section	Chemical compounds	Reference
Leaf	Lupeol-3-acetate, AG, Myricetin-3-*O*-*α*-rhamnoside, Laricitrin-3-*O*-rhamnoside, 3-oxoadipic acid, 3,4-dihydrohybenzoic acid, 3-*O*-galloylquinic acid, 3-glycogallic acid, succinic acid, malic acid, adipic acid, salicylic acid, vanillic acid, GA, caffeic acid, ferulic acid, syringic acid, chlorogenic acid, afzelechin, epicatechin, myricetin, leucodelphinidin, quinic acid, theronic acid, erythordiol, and oleanolic acid.	(30, 31)
Fruit (Pulp and peel)	4-*O*-galloylchlorogenate, 4-*O*-galloylchlorogenic acid, methyl chlorogenate, dihydromyricetin, QUE, t, myricitrin, AG, myricetin-3-*O*-α-L-rhamnoside, L-arabinose, 3-*O*-acyl-L-rhamnose, 3-*O*-acetyl-D-methylgalacturonat, (+)-catechin, (−)-epicatechin, (+)-gallocatechin, GA, protocatechuic acid, resorcinol, 4-hydroxybenzoic acid, vanillic acid, 3′-caffeic acid, 5′-caffeic acid, syringic acid, coumaric acid, ferulic acid, leucoanthocyanidins, leucodelphinidin, leucocyanidin, leucopelargonidin, kaempferol, lutein, zeaxanthin, *β*-cryptoxanthin, lycopene, α-carotene, *β*-carotene, 5, chlorogenic acid, *p*-hydroxybenzoic, ellagic acid, citric acid, fumaric acid, gluconic acid, glyceric acid, glycolic acid, lactic acid, maleic acid, malonic acid, malic acid, oxalic acid, succinic acid, phosphoric acid, quinic acid, benzoic acid, and *trans*-cinnamic acid.	(3, 32–34)
Seed	Β-amyrin, oleanolic acid, lupeol, betulinic acid, D-quercitol, pentacyclic triterpenoid saponin, stigmasterol-3-*O*-*β*-D-glucopyranoside, alkaloids, flavonoids, saponins, tannins, and phenolic compounds.	(3, 34)
Bark	Spinasterol, taraxerol methyl ether, 6-hydroxyflavanone, (+)-dihydrokaempferol, 3,4-dihydroxybenzoic acid, taraxerol. Saponins, tannins, flavonoids, phenolic compounds, alkaloids, steroids, terpenoids, (+)-dihydrokaempferol.	(34, 35)

### 2.1. Extraction conditions

**Figure 3:**
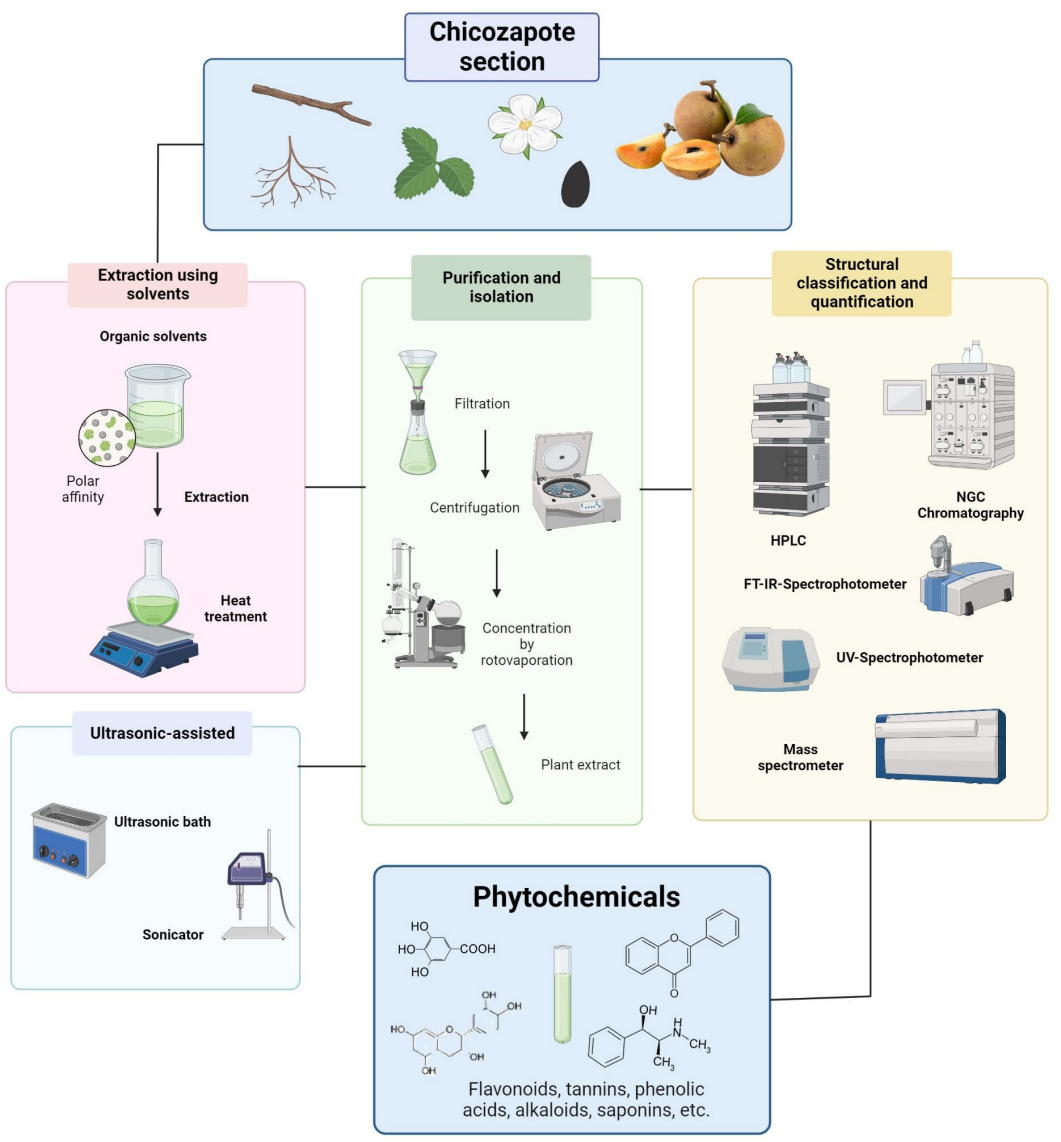
Methodologies for phytochemical extraction on chicozapote sections.

A proper phytochemical extraction methodology is fundamental for the maintenance of biomolecule activity and structure (41). Because organic compounds are only a small part of the total plant mass, the methods used for their extraction, purification, and identification are crucial. Extraction is the first step; it is highly dependent on the nature of the sample matrix and physiochemical properties of the target compounds (33). Several parameters could play important roles in extraction efficiency, such as solvent, temperature, time, pH, particle size of raw materials, and solid–liquid ratio (SLR) (42). Defining the proper parameters and extraction technique improve extraction efficiency, in terms of bioactive compound recovery, yield, time consumption, etc. (33, 42), as has been done with Chicozapote extraction models depicted in Table 2 and Figure 3. Woo et al. (50), proposed an extraction model with several parameters to optimize the antioxidant yields in peel and pulp extracts. These parameters were solvent concentration (Ethanol 0–100%), extraction time (1–5 h), and temperature (25 to 60°C). Using TPC and total antioxidant content (TAC) as parameters evaluation. 2,2-diphenyl-1-picrylhydrazyl (DPPH) free radical scavenging, 2,2′-azino-bis (3-ethylbenzthiazoline-6-sulfonic acid) (ABTS), ferric-reducing antioxidant power (FRAP), and β-carotene bleaching assays were applied. According to the results, in pulp extracts, optimal determined conditions were 40% ethanol, 4 h, and 60°C, with TPC 3.89 ± 0.01 mg (GAE)/g of lyophilized extract. This suggests that most antioxidant compounds are semi-polar. On the other hand, for peel extracts, 80% ethanol, 2 h, and 40°C, TPC 9.23 ± 0.13 mg GAE/g. Karle et al. (49), optimized the extraction conditions to maximize α-glucosidase inhibition and free radical scavenging potential of chicozapote peel extracts. Using an orbital incubator shaker and ice bath was crucial for controlling temperature during the extraction process. Here, optimal parameters were 70% ethanol at 50°C for 12 h. 70% showed the highest half maximum inhibitory concentration (IC_50_) value in the DPPH assay (0.34 mg/ml) and the highest inhibition percentage in the H_2_O_2_ scavenging assay (65.78% at 50 μg/ml).

**Table 2:** Chicozapote extraction yields under different extraction conditions.

Source	Solvent	Yield % per sample (w/w)	Specific extractions conditions	Reference
Leaves	Water	20.70	Dried leaves, 24 h, 2:0.5 SLR, RT	(43)
	Methanol	20.10		
	Ethanol	7.12	80%, dried leaves, 1:5 SLR	(44)
	Petroleum ether	4.41	Dried leaves, 1:10 SLR, muslin cloth	(45)
	Chloroform	4.09	Dried leaves, 2 h, 60 ± 5°C	(46)
	Ethyl acetate	1.45	Dried leaves, 1:10 SLR, muslin cloth	(45)
Pulp	Methanol	39.70	Dried pulp, 24 h, 2:0.5 SLR, RT	(43)
	Water	34.00		
	Ethanol	47.95	Dried pulp, 3 days, 1:10 SLR, RT	(37)
	Ethyl acetate	25.67		
	Hexane	12.61		
	Ethanol	14.02	60%, fresh pulp, 24 h, RT	(47)
		12.76	60%, dried pulp, 24 h, RT	
		12.76	95%, fresh pulp, 24 h, RT	
		11.72	95%, dried pulp, 24 h, RT	
	Ethanol	11.09	100%, fresh pulp, 4 days, RT	(48)
Peel	Ethanol	24.27	Dried peel, 3 days, 1:10 SLR, RT	(37)
	Ethyl acetate	10.21		
	Hexane	4.21		
	Water	19.25	70%, dried peel, 12 h, 1:10 SLR, 28°C Dried peel, 12 h, 1:10 SLR, 28°C	(49)
	Ethanol	18.90		
	Acetone	11.48		
	Chloroform	10.98		
	Hexane	7.50		
Seeds	Ethyl acetate	11.40	Dried peel, 3 days, 1:10 SLR, RT	(37)
	Ethanol	7.14		
	Hexane	5.20		
	Methanol	7.30	Dried seeds 24 h, 2:0.5 SLR, RT	(43)
	Water	5.40		
Flowers	Water	9.40	Dried flowers, 24 h, 2:0.5 SLR, RT	(43)
	Methanol	6.70		
Roots	Water	11.60	Dried roots, 24 h, 2:0.5 SLR, RT	(43)
	Methanol	11.00		
Bark	Ethanol	1.93	Dried bark, 3 days, RT	(35)
	Ethyl acetate	1.86		
	Methanol	14.50	Dried bark, 24 h, 2:0.5 SLR, RT	(43)
	Water	12.50		
Wood	Methanol	18.80	Dried wood, 24 h, 2:0.5 SLR, RT	(43)
	Water	10.60		

Non-conventional, or “green” methods, include techniques such as ultrasound-assisted, microwave-assisted, supercritical fluid, high hydrostatic pressure, among others (42), from which some techniques have been performed on chicozapote models. Ma et al. (31), conducted a comparative study by testing different extraction methods in chicozapote leaves to maximize TFC and TPC. Heat reflux extraction (HRE), ultrasonic-assisted extraction (UAE), and negative pressure cavitation extraction (NPCE) were tested. Optimum parameters were defined through single-parameter experiments: HRE, extract temperature 70°C, 50% ethanol, SLR 30:1, and extraction time of 2 h. HRE allowed the variation of temperature, and the extraction yield for TFC and TPC were the highest with this method. Furthermore, an efficient deep eutectic solvent (DES)-based ultrasound-assisted extraction method was applied in chicozapote pulp extraction. Different DESs have been prepared using carboxylic acids, polyols, and amides. Results indicated that the high polarity and low pH of acid-and amide-based DES enhanced the recovery of polyphenols. Oxaline was the best DES, improving the antioxidants stability and enhancing its antimicrobial activity (30). Ma et al. (43), found that compound isolation could be improved by fractioning an extract. Fresh pulp of chicozapote was extracted twice with methanol at RT, and partitioned by chromatography. The fractions were separated to yield different compounds, including gallocatechin, gallic acid, catechin, and epicatechin, methyl 4-*O*-galloylchlorogenate, galloyl chlorogenic acid, myricitrin, QE, and methyl chlorogenate.

## 3. Mechanisms for biological activity and medicinal applications

Phytotherapeutics are explored due to their uncountable development possibilities. Other advantages include lower costs and side effects incidence (29, 51, 52). Further than the antioxidant activity, these phytochemicals are responsible for most biological activities in plants. Different biological activities have been identified in different structures of chicozapote, including anti-inflammatory, cytotoxic, antimicrobial, anti-hyperglycemic, analgesic, and antinociceptive effects. Furthermore, anti-hypercholesterolemic, hepatoprotective, antiaging, neuro-depressant, and anti-HIV effects have been reported (3, 6, 28) (Table 3). Chicozapote has proved to be safe for human consumption, with a half lethal dose (LD_50_) greater than 2,000 mg/kg (36). In addition, Ganguly et al. (58, 59), reported a lack of side effects or allergic reactions after consumption. These results indicate that chicozapote could be a safe and versatile ingredient for phytotherapeutic formulation.

**Table 3:** Biological activities and applications of different chicozapote extracts.

Biological activity	Application	Source	Assay	Parameter	Dose	Results	Reference
Antioxidant	Antioxidant capacity	Ethanolic pulp extract	DPPH assay ABTS assay	IC50 (μg/mL)	DA	37.63 ± 1.18 73.14 ± 2.84	(48)
Anti-inflammatory	Chronic inflammatory conditions	Methanolic bark extract	Carrageenan-induced edema test Histamine-induced edema test	Right hind paw volume (mm)	200 mg/kg 400 mg/kg 200 mg/kg 400 mg/kg	0.84 ± 0.04 0.58 ± 0.07 0.94 ± 0.03 0.063 ± 0.07	(51)
Anti-inflammatory and cytotoxic	Inflammatory conditions	AG isolated from ethyl acetate leaf extract	3-[4,5-dimethylthiazol-2-yl]-2,5 diphenyl tetrazolium bromide (MTT) assay Griess assay (10 and 25 μg/ml) ELISA (10 and 25 μg/ml)	Cell viability (%) Inhibition of NO production (%), IC50 (μg/mL) Inhibition of PGE2 production (%), IC50 (μg/mL)	10 to 100 μg/mL 10 μg/mL 25 μg/mL 10 μg/mL 25 μg/mL	< 90% viability in 10 to 100 μg/mL 48.05, 65.23 ± 0.41 62.67, 65.23 ± 0.41 34.92, 76.09 ± 0.72 47.51, 76.09 ± 0.72	(39)
Cytotoxic and antitumor	Cancer	Aqueous leaf extract	3-(4,5-dimethylthiazol-2-yl)-2,5-diphenyltetrazolium (MTT) assay (1) Lactate dehydrogenase (LDH) assay (2)	IC50 (μg/mL)	1.56 to 200 μg/mL	HT-29: 42.48 ± 7.40 (1), 46.98 ± 1.23 (2) HCT-116: 92.15 ± 7.89 (1), 88.37 ± 8.23 (2) HeLa: 48.72 ± 3.53 (1), 63.46 ± 6.93 (2) HGT-1: 47.57 ± 5. 12 (1), 91.92 ± 6.54 (2) HepG2: 49.03 ± 5.98 (1), 48.97 ± 7.94 (2)	(53)
Antilipidemic and antidiabetic	Metabolic syndrome	Ethanolic bark extract	*In vitro* α-glucosidase inhibition assay Anti-hyperglycemic assay	Inhibition (%), IC50 (μg/ml) Oral glucose tolerance test and blood glucose (mg/dl) Body weight (g) High-density lipoprotein cholesterol (HDL-c), Low-density lipoprotein (LDL), Total cholesterol, Triglyceride (mg/dl)	100 μg/ml 250 mg/kg 250 mg/kg 250 mg/kg	63.58 ± 1.05, 119.79 ± 1.52 115.68 ± 4.05, 198.68 ± 9.52 6.93 ± 4.83 71.63 ± 2.84, 53.24 ± 2.08, 146.67 ± 4.29, 140.68 ± 5.46	(33)
Antimicrobial and antifungal	Infectious diseases	Ethyl acetate stem bark extract	Disk diffusion assay Serial dilution technique	Zone of inhibition (mm) for antibacterial and antifungal Minimum inhibitory concentration (μg/ml)	300, 600, and 900 μg/disk	Inhibition in all pathogenic bacteria, 8–15 mm. 8-13 mm on *Aspergillus flavus, Fusarium* sp*.,* and *Vasianfactum* sp.	(54)
Antimicrobial synergic effect	Multi-drug resistant microorganisms (MDR)	Methanolic *Cassia fistula* flower and *Chicozapote* leaf extract (1) Ethanolic *Cassia fistula* flower and *Chicozapote* leaf extract (2)	Well-diffusion assay	Zone of inhibition (mm) for antibacterial and antifungal	250–1,000 mg/ml	Inhibition in *Staphylococcus aureus*, 12 to 21 mm (1), and 11 to 21 (2). Inhibition in *Aspergillus niger*, 10 to 21 mm (1), and 11 to 23 mm (2).	(52)
Anti-aging activity	Aging	Ethanolic pulp extract	DPPH assay Collagenase inhibition assay Elastase inhibition assay	IC50 (μg/ml) Collagenase inhibition (%) Elastase inhibition (%)	100 mg/mL 140 μg/mL 80 μg/mL	80.00 ± 1.88 64.66 47.74	(47)
Gastroprotective	Ulcerative colitis	Ethanolic bark extract	Dextran sulfate sodium (DSS) induced colitis Myeloperoxidase (MPO) and Malondialdehyde (MDA) level Catalase (CAT), Sulfur dioxyfenase (SDO), Glutathione (GSH)	Disease activity index (% protection) Weight of Colon (mg/cm) OD/g tissue μg/50 mg tissue	150 mg/kg 200 mg/kg 150 mg/kg 200 mg/kg 150 mg/kg 200 mg/kg	5.47 ± 0.61, 185.28 ± 5.61 3.28 ± 0.57, 170.11 ± 5.11 38.84 ± 2.63, 62.10 ± 3.10 29.43 ± 1.24, 50.21 ± 2.92 34.28 ± 1.60, 58.39 ± 2.55, 27.55 ± 1.61 41.92 ± 2.42, 70.11 ± 2.97, 32.62 ± 1.75	(55)
Hepatoprotective	Hepatic disease	Ethanolic bark extract	DPPH assay CCl4 assay	IC50 (μg/ml) Serum glutamic-oxaloacetic transaminase (SGOT) and Serum glutamic-pyruvic transaminase (SGPT) (U/L) Alkaline phosphatase (ALP) (KA) Total bilirubin and total protein (mg/dl)	500 μg/ml 300 μg/ml 300 μg/ml 300 μg/ml 300 μg/ml	16.83 44.62 ± 0.51, 66.54 ± 1.07 47.70 ± 0.02 4.47 ± 0.02, 8.54 ± 0.08 7.31 ± 0.35	(56)
Anti-arthritic	Ethanolic leaf extract	Arthritis	*In vitro* inhibition of protein denaturation	Protein denaturation (%)	100 μg/ml 250 μg/ml	58.89 ± 6.29 75.84 ± 2.31	(57)
Neuro-depressant	Ethanolic leaf extract	Insomnia	Phenobarbitone-induced sleeping time test	Sleeping time (min)	200 mg/kg	93	(41)
Anti-HIV	Prenylated coumarins isolated from ethanolic pulp extract	HIV	Cytopathic inhibition effects of HIV-1 assay	Half maximal effective concentration (EC50) (μM)	DA	0.12–8.69d	(46)

*DA, does not apply.

### 3.1. Antioxidant activity

Chicozapote is an excellent source of antioxidant. Research published by Ma et al. (43), reported through ABTS assay that chicozapote fruits had an ascorbic acid equivalent antioxidant capacity (AEAC) of 3,396 ± 387.9 mg/kg. This capacity is mainly attributed to basic blocks structures of gallocatechin, catechin, or both (32); which decreases as ripening progresses, along with, TAC and TPC (36). Out of 10 antioxidant compounds isolated from methanolic fruit extract, methyl-4-*O*-galloylchrologenate displayed the highest antioxidant capacity in DPPH assay, IC_50_ = 12.9 μM; followed by 4-*O*-galloylchlorogenic acid, IC_50_ = 23.5 μM (32). Islam et al. (60), tested the antioxidant activity of a 100 μg/ml dose of ethanolic leaf through FRAP and cupric-reducing antioxidant capacity (CUPRAC) assays. Results demonstrated strong reducing potential with 53.3 ± 2.85 and 40.09 ± 3.61 μM AEAC, respectively, which suggests a significant electron-donating capacity. Ethanolic bark extract also showed antioxidant capacity in DPPH assay with IC_50_ = 16.83 μg/ml, compared to ascorbic acid IC50 = 4.87 μg/ml (44). Aqueous and methanolic extracts of different chicozapote structures were evaluated to determine their antioxidant activities. Crude methanolic extract of flowers showed the highest DPPH and ABTS, IC50 = 22.74 ± 0.67, 20.89 ± 0.17 μg/ml, respectively; followed by the aqueous crude extracts of the fruit and roots with an IC50 = 33.08 ± 0.26, 44.24 ± 0.49 μg/ml in DPPH, and 35.29 ± 0.58, 41.34 ± 0.65, respectively. Additionally, in FRAP the results coincided with those of the previous antioxidant assays. The fresh pulp extract exhibited a stronger DPPH potential than dried fruit, suggesting that the drying process can affect antioxidant potential (28). Flavonoids are polyphenols that have significant antioxidant activity through direct scavenging of free radicals, followed by the inactivation and inhibition of lipid peroxidation by metal ion chelation (61). This is possible because of a number of hydroxyl groups present that act as hydrogen-donors (44). QE, myricetin, and myricitrin are flavonoids found in various plants including chicozapote. Sadžak et al. (61), evaluated the capacity of these compounds to resist lipid peroxidation. Despite being an antioxidant, myricitrin exhibits the most potent activity; as it is located closer to the surface of the membrane and is the most hydrophilic, it is more exposed to upcoming radicals and can reach them before they locate a reactive site in the membrane. Therefore, it is capable of inhibiting lipid peroxidation in the phospholipid bilayer membranes. Chicozapote juice also showed antioxidant capacity, potentially inhibiting free-radical lipid peroxidation in a liposome model (32). Although most flavonoids have antioxidant capacity, catechins and flavones provided the strongest protection against reactive oxygen species (ROS) (61).

### 3.2. Anti-inflammatory activity

**Figure 4:**
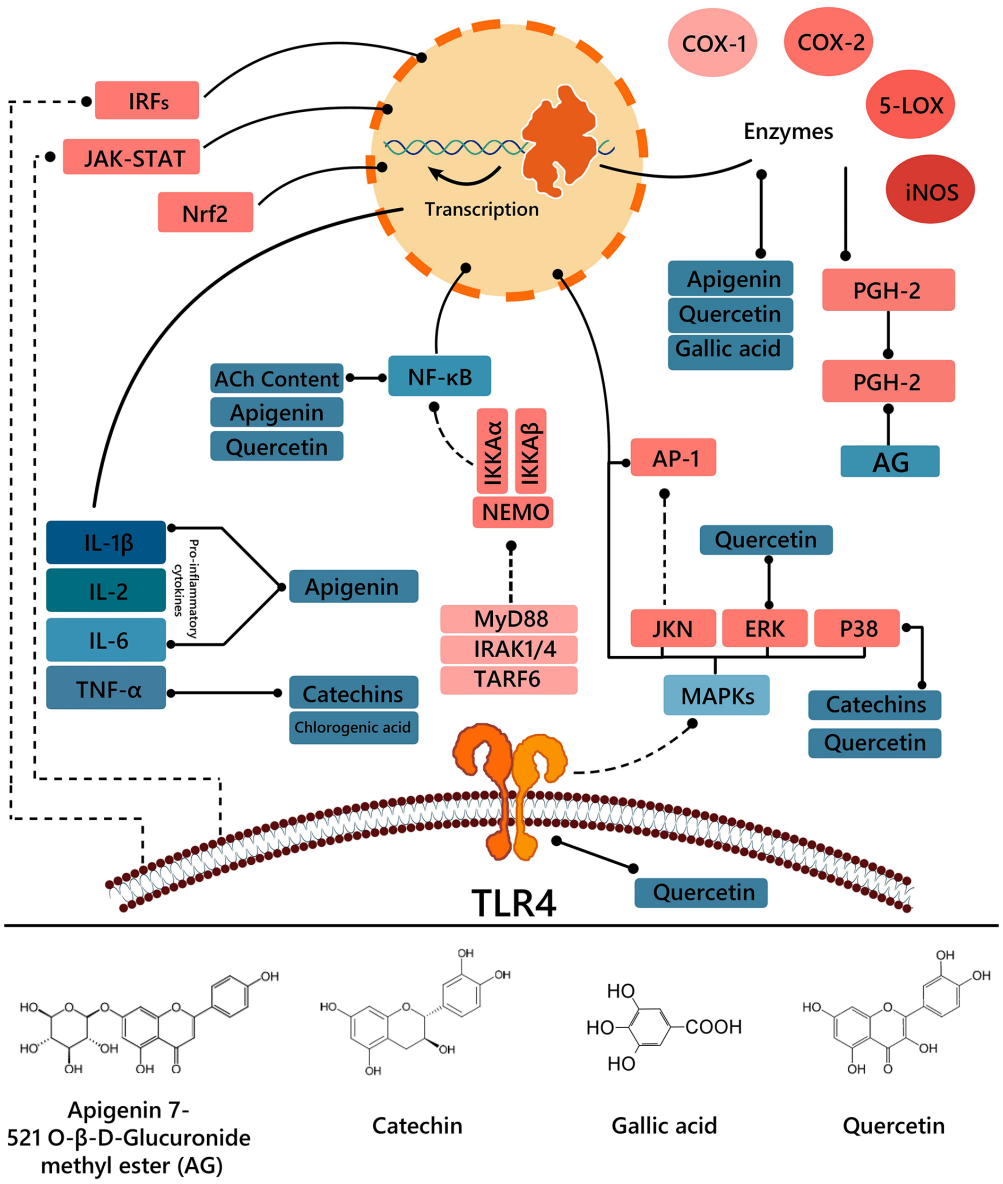
Anti-inflammatory pathway of isolated compounds from chicozapote.

Chronic inflammation stimulates the synthesis of tumor necrosis factor α (TNF-α) and binds to the TNF-α receptor, which is a cyclic inflammatory process (62). On a study performed by Ganguly et al. (58), ethanolic leaf extract of chicozapote exhibited an anti-inflammatory activity in a time-dependent manner. After reaching its peak at 6 h, performance was better than the standard drug, diclofenac sodium100 mg/kg, with 92.75% inhibition. The extract effect was more significant in the second phase, confirming its action mechanism as a prostaglandin (PG) reductive agent, synthesis-inhibiting enzymes of the cyclooxygenase pathway, which are produced during the inflammatory responses. Similarly, Konuku et al. (63), suggested that the ethyl acetate leaf extract anti-inflammatory effect is due to the inhibition of inflammatory enzymes cyclooxygenase-2, 5-lypooxygenase, and phospholipase-2 (COX-2, 5-LOX, and PLA-2). This effect could be due to the presence of QE in the extracts, which was used as a standard drug for 5-LOX inhibition (IC_50_ = 4.851 μg/ml), action mechanisms have also been elucidated. It is known to negatively regulate liposaccharide-induced toll-like receptor 4 (TLR4) expression and signaling, preventing nuclear factor kappa-light-chain-enhancer of activated B cells (NF-κB) translocation and signaling pathway activation of the Nrf2 signaling Cascade; and inhibit COX-2 and inducible nitric oxide synthase (iNOS) expression. It also reduces pro-inflammatory cytokine productions by suppressing extracellular signal-regulated kinase (ERK) activation and p38 mitogen-activated protein (MAP) kinase (64). Hossain et al. (65), concluded through histamine-induced paw edema that chicozapote methanolic bark extract has antihistamine activity with a dose-dependent behavior. Confirming that their mechanism is mediated through the inhibition of inflammatory mediators and can be attributed to a high flavonoid content, particularly QE. GA is also a dominant compound in chicozapote, whose anti-inflammatory response is suggested to interfere with polymorphonuclear leukocyte function (37). Liu et al. (66), reported anti-inflammatory effects of a 90% from chicozapote ethanolic fruit extracts. Obtained results indicated a remarkable decrease in nitric oxide (NO) production, this was observed in mouse macrophage RAW 264.7 cells (IC_50_ = 7.65 ± 0.12 μg/ml). The anti-inflammatory potential of AG, identified in chicozapote phytochemical profile, was assessed. Cells treated with this compound showed decreased production of PG in a dose-dependent manner, as has been seen with other chicozapote extracts, and the anti-inflammatory mechanism is mainly based on the PGE2 synthesis in inhibition (39). As it is rich in catechins, they have been known to improve cholinergic dysfunction by regulating acetylcholine (ACh) content and acetylcholinesterase (AChE) activity in hippocampal tissues. ACh can suppress the expression of NF-κB and inhibit the synthesis of pro-inflammatory cytokines (62) (Figure 4).

### 3.3. Cytotoxic and antitumoral activity

Some phytochemicals of the chicozapote plant exhibit cytotoxic effects over different carcinoma cell lines. Furthermore, several reports claim its safety over normal human cells. Cytotoxic effect of the isolated (+)- dihydrokaempferol from bark extract was determined in human diploid lung fibroblast WI-38. Despite having a potent effect over several cancer cell lines, it was not toxic to the normal cell line WI-38 (>416.22 μM) (38). The cytotoxicity of the ethyl acetate (EtOAc) seed coat extract was evaluated in Vero cells. The EtOAc seed extract was classified as non-toxic in cells at the highest tested concentration (50 μg/ml) (32). Additionally, mouse fibroblast (BALB/c 3T3) cell lines were used as control to evaluate the leaf methanolic extract. The results indicated non-toxic effects (39). The cytotoxicity of EtOAc leaf and stem bark extracts was screened using a brine shrimp lethality bioassay (40). The LC_50_, a 50% lethal concentration μg/ml was determined. Here, ampicillin trihydrate was used as a positive control. Both extracts exhibited cytotoxic effects against *Artermia salina*. Leaf extracts showed a significant effect of LC_50_ = 16.17 μg/ml compared to the positive control LC_50_ = 12.38 μg/ml. In contrast, stem bark extract showed mild activity, LC_50_ = 50.26 μg/ml. This method has proven to be excellent for screening the cytotoxic properties of medicinal plants and has a good correlation with cytotoxic activity in some human tumor cells, as well as pesticidal activity (41). Chicozapote has been shown to exert cytotoxic effects on several cancer cell lines. Islam et al. (60), tested the cytotoxicity of ethanolic leaf extract. Cell viability in mouse myoblasts C2C12 cells was evaluated, exhibiting a moderate to high cytotoxic effect at 100 and 200 μg/ml, with <80% cell viability%. As a potent antioxidant, methyl-4-*O*-galloylchrologenate isolated from chicozapote fruit has also been shown to possess significant cytotoxic activity. Ma et al. (43), reported that this phenolic antioxidant showed a cytotoxic effect on HCT-116 and SW-480 human Colon cancer cell lines, with IC_50_ values of 190 μM and IC_50_ = 160 μM, respectively. In contrast, 4-*O*-galloylchlorogenic acid exhibited cytotoxic effects in HCT-116 and SW-480 cells with IC_50_ = 154 μM and IC_50_ = 134 μM, respectively. Tan and Norhaizan (53) showed aqueous leaf extract is triggers apoptosis and activates the caspase-dependent pathways in several human cancer cells. On agreement with Ma et al. (43), the aqueous leaf extract also probed activity against Colon carcinoma HCT-116 cells in a time-dependent pattern after 24 h with an of IC_50_ = 161.68 ± 5.34 μg/ml until 72 h IC_50_ = 92.15 ± 7.89 μg/ml. It also showed cytotoxic activity in several cancer lines, including colorectal adenocarcinoma HT-29 cells after 24 h until 72 h IC_50_ = 121.20 ± 15.29 and 42.48 ± 7.40 μg/ml, respectively. A similar trend was seen in cervical cancer cell line HeLa, gastric adenocarcinoma HGT-1 cells, and hepatocellular carcinoma HepG2 cell line, after 72 h IC_50_ = 48.72 ± 3.53 and 47.57 ± 5.12, 49.03 ± 5.98 μg/ml, respectively, when cells were inhibited. The cytotoxic effect was further tested in HT-29 cells, which indicated that the aqueous leaf extract triggered morphological changes in cancer cells, induced apoptosis, promoted caspase-3 and caspase-8 activities, decreased MDA levels, elevated catalase levels, and upregulated mRNA levels. Khalek et al. (67), tested the cytotoxicity of an ethyl acetate fruit extract in Ehrlich ascites carcinoma (EAC) cells where doses of 50 mg/kg and 100 mg/kg were administered. Results showed an increase in life span was found to be 41 and 67.7% and a cell growth inhibition of 47.5 and 65.7%, respectively. Rashid et al. (68), used acetate leaf extracts to treat EAC cells; the extracts showed antitumor effects in Swiss albino mice. The treatment decreased tumor cell viability and body weight gain due to the tumor burden. In comparison, Tan and Norhaizan (53) showed that aqueous leaf extracts have cytotoxic effects against the HepG2 cell line. Analyses revealed that the suppression of cancerous cell growth was due to the induction of cell cycle arrest, morphological changes, induction of apoptosis, activation of Bax and downregulation of Bcl-2 proteins, promotion of caspase activity, induction of ROS formation in cancer cells, induction of JNK1 mRNA levels, and inhibition of ERK1/2 and Akt1 response pathways. Tan et al. (69), tested a methanolic leaf extract against HeLa cells. It was concluded that HeLa cells were more sensitive than other cancer cell lines. Overall, the extract inhibited cell viability through the induction of mitochondrial ROS generation and inhibition of NF-κB and epidermal growth factor receptors. Chunhakant and Chaicharoenpong (35) tested isolated compounds from chicozapote bark extracts through a 3-(4,5-dimethylthiazol-2-yl)-2,5-diphenyltetrazolium (MTT) assay. Among the isolated compounds, (+)-dihydrokaempferol demonstrated potent cytotoxicity in the breast carcinoma (BT474), lung bronchus carcinoma cell line (Chago-K1), liver carcinoma (HepG2), gastric carcinoma (KATO-III), and Colon carcinoma (SW620) cell lines. Spinasterol had a significant cytotoxic activity in all the cancer lines tested, whereas 6-hydroxyflavanone showed a moderate effect. On the other hand, taraxerone showed a strong selective effect on some of the carcinoma cell lines (BT474, Chago-K1, HepG2, and KATO-III). From the isolated compounds with a significant cytotoxic activity, only spinasterol exhibited effects on the control cell line (WI-38 lung fibroblast). Furthermore, these compounds displayed anti-tyrosinase activity. Tyrosinase and polyphenol oxidase (PPO) are indirectly responsible for photoaging, resulting in hyperpigmentation. Development of melanoma and other types of skin cancer is due to the generation of ROS, which cause skin pigmentation in melanocytes. Therefore, it is plausible that these isolated compounds could have skin cancer-preventive activity through the inhibition of tyrosinase. On the other hand, several NPs have been synthesized from chicozapote extracts as an antiproliferative agent. Kiriyanthan et al. (70), synthesized Cu NPs using chicozapote leaf extracts. The anticancer activity of the NPs was assessed against breast cancer MCF7 cell lines, and the IC_50_ value was reported to be 53.89 μg/ml, whereas its cytotoxic activity was tested on normal Vero cells, and this resulted in an elevated IC_50_ value of 883.69 μg/ml, indicating that it was not toxic to normal cells. The mechanism of action was also elucidated by acting as kinase inhibitors, causing apoptosis by translocating Bax.

### 3.4. Antilipidemic and antidiabetic activities

Antilipidemic and antidiabetic effects are associated with the different structures of chicozapote. Ethanolic and aqueous leaf extracts showed significant cholesterol-lowering effects in hypercholesterolemic male Wistar rats. The experimental group showed cholesterol levels like those of patients treated with atorvastatin. In contrast, when the extracts were administered to the healthy control group, only the aqueous extract caused a significant decrease. In addition, glucose levels did not decrease in the healthy control group (71). Barbalho et al. (29), evaluated the effects of chicozapote leaves and pulp juice on Wistar rats´ metabolic profile. Rats treated with juice for 50 days had a significantly lower glycemic index than the healthy control group. In contrast, insulin, total cholesterol, and triglyceride levels also decreased in the treated groups compared with those in the healthy control group. Moreover, HDL-c levels were higher in the treated groups than in the control group. However, there were no significant differences between the leaf and pulp juices treatments, except for total triglyceride levels, which were lower in the group treated with pulp juice. Regarding percentage weight gain, the group treated with pulp juice showed a decrease compared to the healthy control group. Polyphenols present in chicozapote (GA, catechin, and epicatechin) have been reported to inhibit pancreatic cholesterol esterases; they can also bind to bile acids, thereby reducing the solubility of cholesterol in the micelles. Recently, the decrease of cholesterol absorption by reducing solubility of cholesterol micellization in the intestinal lumen has become a new alternative to address obesity and hyperlipidaemia (72). GA has been shown to suppress high-fat diet-induced (HFD) dyslipidemia, hepatosteatosis, and ROS. It also decreases body weight gain, liver and adipose tissue weights, serum biochemical parameters, such as LDL-cholesterol, insulin, leptin, phospholipid, total cholesterol, and triacylglycerol, and ROS by enhancing the secretion of antioxidant enzymes (73).

Chicozapote is recognized for its antidiabetic potential. Islam et al. (60), tested the α-glucosidase-inhibitory activity of chicozapote ethanolic leaf extracts. These enzymes hydrolyse carbohydrates into glucose, thereby increasing blood glucose levels. Thus, inhibition of hyperglycaemia in patients with type-2 diabetes may be suppressed. Findings showed a significant inhibition even at 1 μg/ml concentration, with a very low IC50 (2.51 ± 0.15 μg/ml). Chicozapote fruit aqueous extract α-glucosidase IC_50_ was 56 μg/ml, which was much higher than that of the leaf extract. Basal-and insulin-administered glucose uptake improved remarkably by 42.13 ± 0.27%, and 57.74 ± 0.17%, at a 30 μg/ml concentration. On the other hand, acetone fruit extract had a dose-dependent inhibitory effect on α-amylase and α-glucosidase. The inhibitory effect could reach up to 90, at 8 μg/ml, with low IC_50_ values of 4.20 ± 0.20 and 16.6 ± 0.30 μg/ml, respectively (74). Ethanolic peel extract α-glucosidase enzyme inhibition was assessed. The IC_50_ value obtained was 104.23 ± 0.75 μg/mL, which was considered a moderate activity when compared to standard, acarbose (33). Regarding phytochemicals, Wang et al. (74), attributes these activities to proanthocyanidins presence. However, caffeic and chlorogenic acid have a remarkable α-glucosidase inhibition activity too, with data showing a IC_50_ = 4.98 and 9.24 μg/ml, respectively; as well as ferulic acid, epicatechin, and myricetin (60). In addition to phytochemicals, some vitamins in chicozapote fruit exhibit antidiabetic and antiobesity activities. The use of thiamine is related to hyperglycemia prevention because it interferes with both leptin and glycemic levels. Chicozapote fruit decreases leptin and insulin levels, whereas riboflavin reduces oxidative stress and minimizes vascular damage (42).

### 3.5. Antimicrobial activity

Generally, phytochemicals might be more effective against Gram-positive bacteria than against Gram-negative due to morphological differences (45). Peptidoglycan is formed by repeating units of *N*-acetylmuramic acid and *N*-acetylglucosamine residues cross-linked by short amino acid chains. This amino acid sequence plays a crucial role in bacteria protection because it provides strength. Therefore, it is more difficult for phytochemicals to penetrate Gram-negative bacterial cell walls (75). Despite this, chicozapote has shown a broad antibacterial range against clinically important Gram-positive and Gram-negative bacteria (32), meaning it could be effective against a broad spectrum. Osman et al. (54), tested the ethyl acetate extract of chicozapote stem bark on multiple bacteria, i.e., *Bacillus subtilis* BTCC19, *Bacillus megaterium* BTCC18, *Bacillus cereus* ATCC258, *Sarcina lutea* ATCC27803, *Escherichia coli* ATCC25922, *Shigella sonnei* ATCC8992, *Shigella Shiga* ATCC27853, *Shigella dysenteriae* ATCC561, and *Salmonella typhi* ATCC14228; by means of disk diffusion assays. Stem bark extract exhibited moderate activity against all pathogenic bacteria (8 to 15 mm). Antifungal activity has also been reported in an ethyl acetate stem bark extract. Various fungal strains, such as *Aspergillus flavus* ACCT27853, *Aspergillus fumigatus* ATTC10231, *Candida albicans* ATTC25889, *and Vasianfactum* spp. ATTC235561 and *Fusarium spp.* ACCT56390; produced inhibition zones of 8–13 mm for *Aspergillus flavus*, *Fusarium spp.*, and *Vasianfactum* spp. (54). Ganguly and Rahman (76) also determined the antifungal activity of the ethyl acetate fraction of leaf extract and the ethanolic crude extract against different fungi including *Aspergillus niger*, *Candida albicans*, and *Saccharomyces cerevisiae;* with inhibition zones of up to 9 mm. Compared to leaves, seeds have also proven their own activity. The acetone seed extract revealed a minimum inhibitory concentration (MIC) of 400–500 μg/ml in a dose-dependent manner; with a maximum inhibition zone on *Micrococcus luteus* at 500 μg/ml with 16 mm (77). According to Ngongang et al. (78), the methanolic seed extract presented a significant MIC (100 < MIC ≤512 μg/ml) against different *Staphylococcus aureus* strains, including resistant strains SA01, SA39, SA114, MRSA3, MRSA6, MRSA9, MRSA11, and MRSA112.

**Figure 5:**
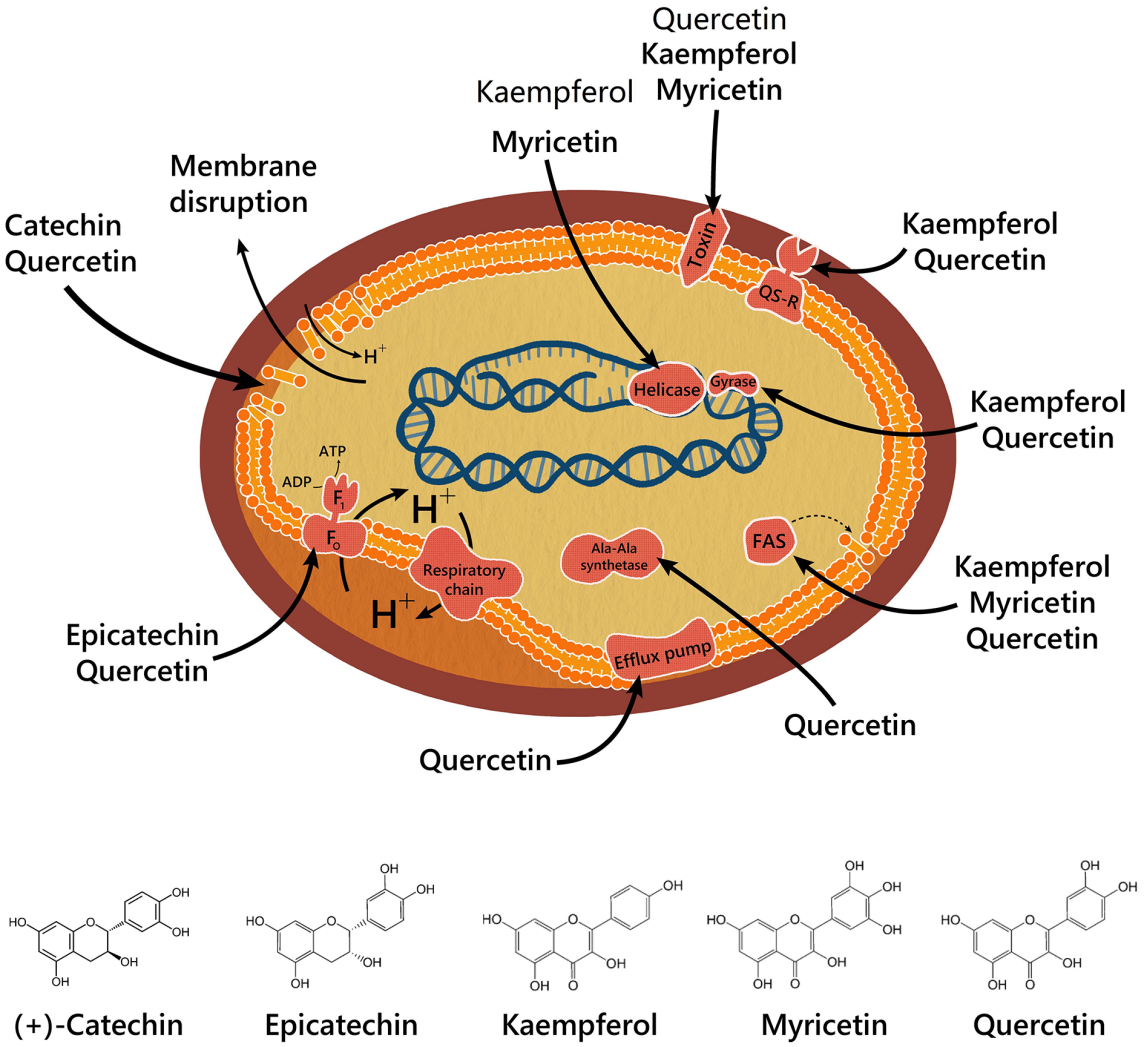
Antibacterial mechanisms from isolated compounds of chicozapote.

Antimicrobial mechanisms of action of some compounds isolated from chicozapote have been elucidated (Figure 5). QE causes GrYB protein secretion, elevation of extracellular phosphatase and β-galactosidase, membrane disruption, ATPase activity inhibition, and inhibition of efflux pumps. Efflux pumps are a major resistance mechanism. Overexpression in resistant bacteria results in the efflux of antibiotics outside the cell, thereby reducing the administrated concentration and rendering the treatment ineffective (79). QE, myricetin, and kaempferol share an inhibitory effect over different element synthesis, including bacterial toxin, cell membrane through FAS-II and Ala-Ala synthase, and DNA by DNA-gyrase mechanism for QE, and helicase mechanism for myricetin and kaempferol. Kaempferol and QE inhibit virulence enzymes through a sortase-inhibition mechanism. (+)-Catechin disrupts the cell membrane, whereas epicatechin blocks ATPase activity. Furthermore, apigenin has shown a range of mechanisms, including membrane disruption and inhibition of biofilm and DNA synthesis (75, 80, 81). Phytochemicals can potentiate the activity of antibiotics on more than 70% of bacteria and could be suggested as potential efflux pump inhibitors (78). Therefore, synergetic relationships between phytochemicals and conventional antimicrobials agents are being explored. Myricetin, QE, and kaempferol, in combination with isoniazid, had a synergistic effect on *Mycobacterium* spp., improving its activity. In contrast, (−)-epicatechin, when combined with tetracycline or isoniazid, had an inhibitory effect on efflux pumps, which are commonly seen as resistance mechanisms in Gram-negative bacteria (82). The synergistic interaction of chicozapote structures has also been tested. Chicozapote pulp, leaf, and seed methanolic extracts exhibited antibiotic-modulating activity in combination with the following six antibiotics: tetracycline, ciprofloxacin, chloramphenicol, erythromycin, kanamycin, and streptomycin; against selected MDR bacteria (50). Meanwhile, Archana and Geetha Bose (83) proved that the combination of *Cassia fistula* flowers and leaves with chicozapote leaves enhanced the antimicrobial activity with a maximum inhibition zone of 23 mm on *Staphylococcus aureus,* and 23 mm on *Aspergillus niger*. Results indicate that a polyherbal extract could improve the efficiency of antimicrobial activity compared with individual plants. As another strategy to maximize the antimicrobial activity of chicozapote extracts, different NPs with antibacterial and antifungal activities have been synthesized from these extracts. Kiriyanthan et al. (70), used chicozapote leaf extract to synthesize Cu NPs to maximize antifungal activity. Otari et al. (84), synthesized silver NPs from aqueous seed extracts and investigated their activity against *Candida* ssp. Shaniba et al. (85), used petroleum ether and acetone seed extracts as sources for silver NPs synthesis, which also proved to have antibacterial activity. Ayodhya et al. (86), used aqueous peel extract to synthesize CeO2 NPs. Among other activities against plagues, Rajakumar and Rahuman (87) tested acaricidal activity of aqueous extract and silver NPs of chicozapote leaves. Kamaraj et al. (88), demonstrated the feeding deterrent activity of synthesized silver NPs and aqueous crude extract from chicozapote leaves against *Musca domestica*. Furthermore, Mourão-Mulvaney et al. (89), proved that isolated chlorogenic acid (200 μg/ml) from the ethanolic leaf extract applied to *Strongyloides venezuelensis* parasites, had a lethal effect on the parasites within 24 h.

### 3.6. Analgesic and antinociceptive activity

Pain is a response to conditions associated with direct tissue damage or indirectly linked to other injuries, including inflammatory processes (49). Analgesic properties were discovered in chicozapote extracts. Jain et al. (90), tested the analgesic effect of petroleum ether and ethanolic leaf extracts using the hot-plate method. Application of thermal stimuli in the hot-plate method causes acute pain mediated through the neuronal pathway, which processes non-inflammatory pain (51). Obtained results indicated that both extracts, at a 200 mg/kg dose, had significant analgesic effects. The increase in reaction time in animals treated with the extracts is an important parameter of Central and peripheral analgesic activity via non-selective COX inhibition and nociceptors. The analgesic activities of methanolic and petroleum ether leaf extracts were evaluated using the acetic acid-induced writhing method. At the evaluated dose of 200 mg/kg, a significant effect was observed, with 94.27 and 96.82% inhibition of acetic acid-induced writhing in mice, respectively (91, 92). Ganguly et al. (58), evaluated the analgesic properties of ethanol, petroleum ether, and ethyl acetate leaf extracts. Acetic acid-induced writhing and radiant heat tail-flick methods were used to evaluate both peripheral and Central antinociceptive activity. The heat tail-flick method measures a complex non-inflammatory response, acute nociceptive input, and is used to study Central antinociceptive activity mediated by the spinal pathway. Results of the acetic acid-induced writhing method indicated that the number of abdominal contractions was significantly reduced in mice treated with 400 mg/kg of ethanolic and petroleum ether extracts, showing 59.89 and 58.24% inhibition, respectively; compared to the positive control (59.34%). Promising potential was also noted for the ethyl acetate extract (46.7%). Furthermore, significant analgesic activity was observed when using the radiant heat tail-flick method. The elongation of the reaction time in mice treated with a 400 mg/kg extract dose of ethanolic and petroleum ether extracts was 88.22 and 52.05%, respectively, 30 min after administration. However, the Central antinociceptive property decreased with time, at 90 min, both extracts increased to 74.15 and 82.15%, respectively (51, 93). In the peripheral antinociceptive mechanism, PG inhibition may be involved (58). Additionally, mechanism may be related to the inhibition of aldose reductase by flavonoid compounds. In contrast, Central analgesic activity may be related to binding of the opioid μ-receptor of the Central nervous system (CNS), which exerts analgesic effects (58). Other opioid receptors may be involved in this effect, such as *k* and δ, indicating that chicozapote may act through these receptors to reduce pain (93). Yong et al. (51), found that chloroform leaf extracts produced a dose–response relationship through the supraspinal pathway during a hot-plate test. In addition, the methanolic leaf extract exhibited analgesic activity. This confirmed the presence of peripherally and centrally acting compounds, indicating that bioactive compounds could differ in their polarity. Catechins; for example, epigallocatechin, (+) catechin, (−) epicatechin, and (+) gallocatechin, chlorogenic acid and its derivatives are recognized for their analgesic effects through the inhibition of TNF-α.

### 3.7. Anti-aging activity

Skin aging is a complex process that results from both intrinsic and extrinsic factors. UV exposure is the most common extrinsic factor, whereas passage of time is the main intrinsic factor for aging. Collagen and elastin are the main proteins involved in maintaining the structural integrity of the skin. Collagenase is responsible for extracellular matrix (ECM) remodeling, including collagen breakdown. The combination of aging factors generates ROS, matrix metalloproteinases (MMPs), and elastase. Thus, aging is associated with oxidative damage. Depletion of collagen and elastin can generate aging signs and wrinkles on the skin (48). *In vitro* anti-aging activities of the chicozapote ethanolic pulp extract have been reported. Chaianuchittrakul et al. (47), reported that 60 and 95% ethanolic fresh pulp extracts had a significant inhibitory activity against collagenase and elastase enzymes, both responsible for skin aging processes. It was revealed that both extracts, at a 140 μg/ml dose, showed a strong inhibition of collagenase enzyme, with 66.42 and 64.66%, respectively. However, they were lower compared to the 20 μg/mL standard dose of epigallocatechin gallate (EGCG), with 98.43%. In contrast, its inhibitory activity against elastase was examined. Of the 60 and 95% extracts, only the 95% showed a significant inhibitory effect. At 80 μg/ml dose, the inhibition was 47.74%, higher than the standard 45.51%. Meanwhile, Pientaweeratch et al. (48), characterized anti-aging activities of 100% ethanolic pulp extract. TFC, TPC, DDPH, *in vitro* collagenase inhibition of MMP-1 and MMP-2, and elastase inhibition assays were performed. The antioxidant activity probed a dose-dependent behaviour, with IC_50_ = 37.65 ± 1.18 μg/ml in DPPH, and IC_50_ = 73.14 ± 2.84 μg/ml in ABTS. Regarding specific anti-aging activity, the inhibition of collagenase and elastase was dose dependent. Despite having an effective collagenase inhibition activity of IC_50_ = 89.61 ± 0.96, 86.47 ± 03.04 μg/ml, it is still 10 times lower than standard EGCG with 9.73 ± 0.18, 8.19 ± 0.40 μg/ml. Nevertheless, the elastase inhibition was three times higher in chicozapote ethanolic extract, IC_50_ = 35.73 ± 0.61 μg/ml, when compared to standard, IC_50_ = 93.99 ± 3.44 μg/ml. This effective anti-elastase activity might be related to the presence of flavonoid compounds, such as QE, myricetin, epicatechin, and catechin, which have all been assessed in previous studies Chicozapote bark has been used to treat hyperpigmentation, which is responsible for photoaging. Chunhakant and Chaicharoenpong (35), isolated anti-tyrosinase compounds using a mushroom tyrosinase inhibition assay. The ethyl acetate bark extract exhibited the highest anti-tyrosinase activity with a IC_50_ = 191.69 ± 6.05 μg/ml, followed by the hexane extract that resulted in IC_50_ = 557.03 ± 24.13 μg/ml, which is a lot less effective. (+)-Dihydrokaempferol was the most potent tyrosinase inhibitor among the isolated compounds. Indicators of tyrosinase inhibitory activity were the monophenolase and diphenolase inhibition, which had IC5_50_ values of 45.35 ± 0.60 and 55.41 ± 0.33 μM, respectively. This compound may have potential as an anti-aging agent and thus may be a preventive agent for skin cancer. Another study tested the anti-tyrosinase activity of different chicozapote structure methanolic extracts, including the roots, flowers, bark, and leaves. The methanolic bark extract probed the highest monophenolase inhibitory activity, IC_50_ = 85.2 ± 2.1 μg/ml, in contrast, the methanolic root extract displayed the strongest diphenolase inhibitory activity, IC_50_ = 33.52 ± 0.68 μg/ml, both anti-tyrosinase indicators. In both extracts, (+)-dihydrokaempferol was detected, and the highest (+)-dihydrokaempferol concentration was found in the methanolic bark extract. This suggests a correlation between anti-tyrosinase activity and this specific compound (28). To determine its antioxidant activity, Kashif and Akhtar (94) developed a natural emulgel sunscreen loaded with chicozapote fruit extract. It was concluded that the formulation was non-irritant and safe for human use; it has promising quenching effects against ultraviolet A (UVA) and UVB radiation. It is important to note that leaves possess a higher elastase inhibitory activity than fruits.

### 3.8. Gastroprotective activity

Antidiarrheal, antiulcer, and antisecretory effects have been studied to determine the mechanism of action of chicozapote fruit regarding these effects. The antidiarrheal effect of the leaves may be due to the inhibition of PG biosynthesis in the intestinal mucosa (58). The fruit exhibited a protective effect against induced diarrhea owing to its relaxing constituent that relieved fluid secretion. This mechanism is believed to be regulated by dual blockade of phosphodiesterase enzyme receptors and Ca^2+^ channels. Moreover, the presence of phytochemicals, such as flavonoids, alkaloids, polyphenols, and saponins, targets gut microbiota activity through metabolites (95). *In vitro*, proteomic, and *in silico* assays were performed with chloroform and aqueous chicozapote fruit extract. Mouse models were used for the *in vivo* assay, were antimotility and antiulcer effects were assessed trough charcoal meal transient time and ethanol-induced ulcer assays; whereas the molecular studied involving proteomic analysis and virtual screening were performed using a discovery studio visualizer (DSV). Both chloroform and aqueous extract exhibited a dose-dependent protection against the induced diarrhea. Gastroprotective effects were observed in ulcerative mouse tissues, along with a decrease in IL-18 levels, as determined by proteomic analysis. Finally, virtual screening results from the *in silico* assay demonstrated consistency against the *in vitro* findings (95). In mouse models, ethanolic bark extract show an antidiarrheal mechanism as well, treatment with bark reduced fecal output by 29.31% and 41.37° at 250 mg/kg and 500 mg/kg doses, respectively (32). Furthermore, the anticholinergic effect on intestinal transit is due to diminished gastrointestinal tract motility tone, which causes an increase in the stay substances in the small intestine, allowing for better water absorption and affecting the peristaltic movement of the intestine. The potential for ulcer treatment may be due to its Ca^2+^ channel blocker (CCB) effect and its role as an active antagonist of Ca^2+^. Antioxidant and NO free radical scavenging may also help to enforce antiulcer activity due to the reduction of oxidative stress in gastrointestinal tissue. Several important anti-inflammatory effects have been previously reported. In rats, chicozapote fruit extract decreased TNF-α, p-NFxB, and COX-2 levels compared to the omeprazole control. It also has a wider therapeutic index since no mortality was observed at a dose of 5 g/kg (95). However, the presence of catechins can regulate the intestinal microbial balance through metabolite production. As discussed previously, catechins absorbed in the blood have important bioactivities; also, unabsorbed catechins also play a significant role. They can act as prebiotics, stimulating the growth and thriving of symbiotic bacteria and protecting against pathogenic bacteria (62). Chicozapote has also been shown to be effective against gastrointestinal disorders. Fruit extracts were evaluated *in vitro* and *in silico* for its purpose in gastric disorders, such as gastritis, constipation, and diarrhea. Bark ethanolic extracts have been reported to be effective when treating ulcerative colitis. The study revealed that MPO activity was strongly inhibited; thus, colonic MDA content decreased. Moreover, the potent antioxidant effect of the extract was proven by restoring normal levels of antioxidant parameters including catalase, SOD, and GSH (55).

### 3.9. Anti-arthritic activity

The effect of chicozapote extracts has been tested as anti-arthritic agents. In an *in vitro* protein denaturation model of rheumatoid arthritis (RA), ethanolic extracts were able to inhibit denaturalization. They found a significant protective effect by using 100 and 250 μg/ml concentrations, reducing protein denaturation to 58.89 and 75.84%, respectively, indicating its potential as an anti-arthritic agent (96). Singh et al. (57), demonstrated the anti-arthritic activity of leaves extracts in rheumatoid arthritis. Ethanolic leaf extract had an inhibitory effect on protein denaturation assay, with 75.84 ± 2.31% of inhibition at a 250 μg/ml dose. Ijaz et al. (97), determined the intrinsic anti-arthritic potential of synthesized nanoparticles (NPs) from leaf extract and gold. The leaf extract showed a significant protection in the protein denaturation model; it also inhibited paw edema by 61.19% on at a 400 mg/kg dose. However, the gold NPs showed greater prominent action compared to the aqueous extract, with an inhibition of 83.34% in Sub-acute arthritis. A decrease in alanine transaminase (ALT), aspartate transferase (AST), and alkaline phosphatase (ALP) activities was also observed, as they play an important role in the formation of biologically active chemical mediators.

### 3.10. Other biological activities

Islama et al. (44), tested the hepatoprotective activity of ethanolic bark extracts through an *in vivo* rat model with a CCl4 assay. Findings indicated restoration of liver marker enzymes SGOT, SGPT, and ALP, total bilirubin, total protein, and liver weight. This restoration can lead to stabilization of the plasma membrane, repair of hepatic tissue damage, and stabilization of endoplasmic reticulum. Furthermore, Islam et al. (56), performed an *in vivo* model to test ethanolic leaf extract capacities. Results indicated that the extract may have hepatoprotective activity against several liver marker enzymes and total bilirubin. Alrashood et al. (16), demonstrated that lyophilized chicozapote extract exhibited protective effects against CCl4 and lipid-lowering activity. The treatment elevated biomarkers for liver damage in a dose-dependent manner, in accordance with the results of other studies. This suggests that several chicozapote structures can exhibit hepatoprotective activity. Ganguly and Rahman (76) demonstrated Central nervous system (CNS) depressant activity using an ethanolic leaf extract. Prolonged sleep time was observed through the phenobarbitone-induced sleeping time test in mice. Using a 200 mg/kg dose, the duration of sleep time significantly increased to 93 min compared to the standard diazepam 101.6 min, and control groups with 33.2 min. This might be due to the presence of compounds that induce sedation by potentiating GABA-mediated postsynaptic inhibition through the allosteric modification of GABA receptors. Moreover, Liu et al. (66), revealed that a 90% fruit ethanol extracts have an anti-HIV effect. According to the inhibition assay for cytopathic effects of HIV-1 (EC_50_), the anti-HIV-1 reverse transcriptase activity was evaluated against C8166 cells. Prenylated coumarins displayed anti-HIV effects with an EC_50_ range of 0.12 8.69 μg/ml. Manizapotin, a coumarin isolated from the extract, exhibited the most remarkable inhibitory effect, with EC_50_ = 0.12 μg/ml. These results suggested that chicozapote is a promising natural agent against HIV.

## 4. Conclusion

*Manilkara zapota* “chicozapote” has proven its value beyond nutrition. Owing to its unique characteristics and flavor, chicozapote is now distributed worldwide, and its consumption has popularized throughout the years. However, high perishability is a disadvantage for the commercialization and development of value-added products. As a preservation method, it is usually transformed into jams, syrup, ice cream, and fruit bars, among other food products. Nevertheless, its phytochemical-specific profile grants the fruit health-beneficial compounds with broad-spectrum bioactivity. Therefore, revalorization uses fruits as a raw material for the design of functional foods and products. Extraction conditions are the first step in conserving phytochemicals and their bioactivity; hence, it is crucial to apply a proper extraction methodology. Chicozapote extracts exhibits antioxidant, anti-inflammatory, cytotoxic, antidiabetic, antimicrobial, analgesic, anti-aging, gastroprotective, hepatoprotective, anti-arthritic, neuro-depressant, and anti-HIV activity. Its versatility and low toxicity suggest that chicozapote is a promising food source for phytotherapeutics, with several medicinal applications. More research is needed to elucidate its specific mechanisms of action to exploit its full potential.

## Author contributions

MR-G: writing—original draft preparation. LG-A and AS-L: writing—review and editing and final approval of the version to be published. RG-V: review and editing. All authors contributed to the article and approved the submitted version.

## Conflict of interest

The authors declare that the research was conducted in the absence of any commercial or financial relationships that could be construed as a potential conflict of interest.

## Publisher’s note

All claims expressed in this article are solely those of the authors and do not necessarily represent those of their affiliated organizations, or those of the publisher, the editors and the reviewers. Any product that may be evaluated in this article, or claim that may be made by its manufacturer, is not guaranteed or endorsed by the publisher.
